# A compartment-based myocardial density approach helps to solve the native T1 vs. ECV paradox in cardiac amyloidosis

**DOI:** 10.1038/s41598-022-26216-9

**Published:** 2022-12-16

**Authors:** Bishwas Chamling, Michael Bietenbeck, Stefanos Drakos, Dennis Korthals, Volker Vehof, Philipp Stalling, Claudia Meier, Ali Yilmaz

**Affiliations:** 1 Department of Cardiology I, Division of Cardiovascular Imaging, University Hospital Münster, Albert Schweitzer Campus 1, A1, 48149 Münster, Germany; 2 Division of Electrophysiology, Department of Cardiovascular Medicine, University of Muenster, Albert-Schweitzer-Campus 1, Building A1, 48149 Münster, Deutschland

**Keywords:** Cardiac hypertrophy, Heart failure

## Abstract

Cardiovascular magnetic resonance (CMR) plays an important clinical role for diagnosis and therapy monitoring of cardiac amyloidosis (CA). Previous data suggested a lower native T1 value in spite of a higher LV mass and higher extracellular volume fraction (ECV) value in wild-type transthyretin amyloidosis (ATTRwt) compared to light-chain amyloidosis (AL)—resulting in the still unsolved “native T1 vs. ECV paradox” in CA. The purpose of this study was to address this paradox. The present study comprised N = 90 patients with ATTRwt and N = 30 patients with AL who underwent multi-parametric CMR studies prior to any specific treatment. The CMR protocol comprised cine- and late-gadolinium-enhancement (LGE)-imaging as well as T2-mapping and pre-/post-contrast T1-mapping allowing to measure myocardial ECV. Left ventricular ejection fraction (LV-EF), left ventricular mass index (LVMi) and left ventricular wall thickness (LVWT) were significantly higher in ATTRwt in comparison to AL. Indexed ECV (ECVi) was also higher in ATTRwt (*p* = 0.041 for global and *p* = 0.001 for basal septal). In contrast, native T1- [1094 ms (1069–1127 ms) in ATTRwt vs. 1,122 ms (1076–1160 ms) in AL group, *p* = 0.040] and T2-values [57 ms (55–60 ms) vs. 60 ms (57–64 ms); *p* = 0.001] were higher in AL. Considering particularities in myocardial density, “total extracellular mass” (TECM) was substantially higher in ATTRwt whereas “total intracellular mass” (TICM) was rather similar between ATTRwt and AL. Consequently, the “ratio TICM/TECM” was lower in ATTRwt compared to AL (0.58 vs. 0.83; *p* = 0.007). Our data confirm the presence of a “native T1 vs. ECV paradox” with lower native T1 values in spite of higher myocardial mass and ECV in ATTRwt compared to AL. Importantly, this observation can be explained by particularities regarding myocardial density that result in a lower TICM/TECM “ratio” in case of ATTRwt compared to AL—since native T1 is determined by this ratio.

## Introduction

Amyloidosis is a multifaceted, heterogeneous disease characterised by pathological accumulation of insoluble, polymeric protein fibrils in the extracellular space of various tissues and organs. Deposition of amyloid fibrils in the myocardium leads to an infiltrative/restrictive cardiomyopathy. The most common forms of amyloidosis infiltrating the human heart (cardiac amyloidosis, CA) are (a) immunoglobulin light chain (AL)^1^ and (b) transthyretin amyloidosis (ATTR)^2^ which in turn comprises two subtypes: a hereditary form (ATTRv) caused by the presence of a TTR gene mutation and a wild-type form (ATTRwt) caused by age-related instability of wild-type TTR^3^.

Cardiovascular magnetic resonance (CMR) is a robust diagnostic tool enabling detailed work-up of different cardiac diseases including LV hypertrophy of unknown origin. CMR was recently shown to offer a very high diagnostic yield regarding the diagnosis of cardiac ATTR and AL amyloidosis^4,5^. Moreover, the severity of cardiac involvement in amyloidosis can be assessed by measurement of LV wall thickness, LV mass and the degree of late gadolinium enhancement (LGE) in CMR^6–8^. In addition, CMR techniques such as LGE-imaging^7,9^, pre-/post-contrast T1-mapping with subsequent ECV calculation^10,11^ and feature tracking (FT) for strain analysis ^12^ allow to depict both the pattern and the extent of CA^8,13–15^.

Previous CMR studies addressing both ATTRwt and AL patients suggested a lower native T1 value in spite of a relatively higher LV mass and higher ECV value in ATTRwt compared to AL—resulting in the “native T1 vs. ECV paradox” in cardiac amyloidosis that is still unsolved^16–19^. In the present study, we analysed multi-parametric CMR data of ATTRwt and AL patients in order to independently test and verify the presence of the “native T1 vs. ECV paradox” in cardiac amyloidosis. Moreover, we tried to find out an explanation for this “native T1 vs. ECV paradox” based on our comprehensive, multi-parametric CMR data and by considering particularities in myocardial tissue composition and density.

To better understand our novel approach, we need to make us aware of LV mass calculations by CMR: they are based on a pre-specified “myocardial density” factor (usually 1.055 g/ml) and allow to calculate LV “mass” based on a contoured 3D volume of the LV. Importantly, this approach does not consider any potential differences in “myocardial density” or “gravity”, caused by e.g. the presence of specific cardiomyopathies (such as cardiac amyloidosis) … nor does it account for any differences in myocardial density between the intracellular vs. extracellular space of the myocardium. Considering the fact that amyloid fibrils accumulate in the “extracellular” space of the myocardium only, the undifferentiated use of the aforementioned myocardial density factor of “1.055 g/ml” for volume-based calculations of myocardial mass are neither appropriate nor acceptable. Therefore, we tried to address this issue by a different approach.

## Methods and materials

### Patient characteristics and study design

In the present single-centre study, we carefully analysed the clinical, laboratory and CMR imaging data of 120 amyloidosis patients who were not treated with any disease specific therapy prior to our CMR study. All patients underwent comprehensive cardiac work-up including a multi-parametric CMR study and only patients with biopsy and/or bone scintigraphy-based proof of cardiac involvement were included. Cardiac biomarkers (N-terminal pro brain natriuretic peptide, NT-proBNP) as well as renal parameters (eGFR, estimated glomerular filtration rate) were obtained on the day of CMR at our specialised “cardiac amyloidosis unit” at the University Hospital Muenster, Germany.

All patients enrolled were assigned into two groups: (a) ATTRwt amyloidosis (79 male/11 female, 79 ± 5 years) and (b) AL amyloidosis (20 male/10 female, 66 ± 8 years) on the basis of established diagnostic algorithms (comprising e.g. immunofixation, bone scintigraphy, endomyocardial biopsy)^14^. All patients with AL amyloidosis showed presence of elevated monoclonal proteins with elevated free light chains either in blood, urine or both. The other group of patients enrolled had either histologically proven ATTR and/or positive bone scintigraphy (in addition to positive CMR) in the absence of monoclonal gammopathy. A hereditary form of ATTR (ATTRv) was ruled out by genetic analysis and only patients with wild-type ATTR (ATTRwt) were included.

### Ethics approval and consent to participate

Written informed consent was obtained from all the patients for the publication of any images or data included in this article. The study protocol is in accordance with the ethical guidelines of the 1975 Declaration of Helsinki and with the laws and regulations of Germany. The protocol was approved by the local ethics committee (Ethikkommission der Ärztekammer Westfalen-Lippe) of the University Hospital Muenster, Germany (ID 2019-437-f-S).

The study protocol complies with the Declaration of Helsinki. Ethics approval was obtained from local authorities and written informed consent was obtained from the participants.

### CMR acquisition, T1 and ECV measurement

CMR studies were performed on a 1.5-T system (Ambition, Philips Healthcare, Best, The Netherlands). CMR data acquisition was performed according to the standardized protocols^20^. Our CMR protocol comprised a cine steady-state free precession pulse sequence for ventricular function and a two-dimensional (2D) inversion recovery fast spoiled gradient-echo sequence 10-15 min after administration of a gadolinium-based contrast agent (Gadobutrol 0.15 mmol/kg) for detection of myocardial pathology as described earlier^21^. Image analysis and interpretation was performed using commercially available software (cvi42 version 5.12.0, Circle Cardiovascular Imaging, Calgary, Alberta, Canada). Analysis of ventricular volumes and function as well as LV mass was made by contouring short-axis cine images.

In addition, a modified Look-Locker inversion recovery (MOLLI) T1-mapping sequence was applied in basal, mid and apical short-axes prior to contrast agent administration and ~ 20 min thereafter to determine native T1 and ECV values as described previously^21^. Motion corrected native and post-contrast T1-maps were generated from the pre- and post-contrast T1-sequences. Motion corrected and segmented ECV maps were generated from the native and post-contrast segmented T1-maps, using the patient’s haematocrit level, measured on the same day as described by us elsewhere^21^. For T2-mapping, similar to T1 mapping, motion corrected but only native pre-contrast sequences were used. “Global” T1, T2 and ECV values were calculated by averaging all 16 segments from three short-axis slices.

In addition, (a) relative intracellular volume (ICV) was calculated with the formula ICV = [1–ECV]; (b) “total intracellular mass” (TICM) was calculated with the formula TICM = [ICV × total LV myocardial volume × myocardial density] and (c) “total extracellular mass” (TECM) with the formula TECM = [ECV × total LV myocardial volume × myocardial density] as described in previous works^18,22^. Noteworthy, when calculating TICM, we used the well-known myocardial density value for humans of 1.055 g/ml. However, when calculating TECM, we used a different myocardial density value of 1.38 g/ml, since this value is expected to be more appropriate in case of extracellular “protein” accumulation—as is the case in an extracellular infiltrative disease such as cardiac amyloidosis^23^.

### Feature tracking analysis

For the assessment of global LV deformation, three-dimensional (3D) LV global longitudinal strain (LV-GLS) derived from feature tracking (FT) was obtained using a validated algorithm integrated in the analysis software^24^. Landmarks for LV base (at the mitral valve ring) and apex were defined at end-diastole in all long-axis slices. Endocardial and epicardial borders were manually contoured in the end-diastolic frame in the three long-axis slices and in three short-axis slices, the most basal slice without through-plane distortion from the LV outflow tract, a mid-ventricular and an apical slice. Both the landmarks and the contours were automatically propagated throughout the cardiac cycle and manually corrected in case of inaccuracies. Subsequently, relative apical longitudinal strain (LS) was calculated based on the following equation: average apical LS / (average basal LS + mid LS), as defined by Phelan et al.^25^.

### Statistical analysis

As all data were non-normally distributed and therefore, non-parametric tests were used. Most variables are presented as medians and interquartile ranges (median ± interquartile range), few other parameters like ejection fraction (EF) and strain are expressed as change (%) from baseline (BL)—also mentioned in Tables 1 and 2. Differences between groups were calculated with the Mann–Whitney *U* test. Statistical analysis was performed with SPSS (version 27.0, IBM Corp., Armonk, NY). A *p*-value < 0.05 was considered statistically significant. All figures were drawn by Prism Version 8 (GraphPad Software, La Jolla, USA).

**Table 1:** Baseline patient characteristics of wtATTR and AL patients.

Parameter	ATTRwt	*n*	AL	*n*	*p* value
Age (years)	80 (76–83)	90	67 (60–70)	30	< 0.001
Males / females	79 / 11	90	20 / 10	30	< 0.001
BMI (kg/m^2^)	26 (24–29)	90	24 (22–28)	30	0.044
eGFR (CKD-EPI) (mL/min/1.73 m^2^)	58 (45– 60)	90	58 (33–60)	30	0.59
NYHA class	2 (1.0–3.0)	90	2.0 (1.0–3.0)	30	0.34
NT-proBNP (pg/ml)	2,164 (1,293–4,116)	90	2,863 (1,375–5,271)	30	0.33
Major CMR findings					
LV-EF (%)	52 (47–58)	90	58 (50–61)	30	0.012
LV-EDVi (ml/m^2^)	86 (76–97)	90	75 (65–88)	30	0.003
LV-ESVi (ml/m^2^)	39 (32–50)	90	32 (26–40)	30	0.010
LV mass index (g/m^2^)	91 (79–105)	90	81 (61–98)	30	0.004
Max. LV thickness (mm)	19 (18–21)	90	16 (14–18)	30	< 0.001
RV-EF (%)	52 (46–56)	90	57 (46–64)	30	0.11
RV-EDVi (ml/m^2^)	83 (69–100)	90	73 (64–82)	30	0.006
RV-ESVi (ml/m^2^)	40 (30–52)	90	34 (24–39)	30	0.019
3D global longitudinal peak strain (%)	-8.0 (-9.38 to -6.15)	85	-7.2 (-9.98 to -6.18)	30	0.68
Apical / (basal + mid) strain ratio (3D), n	0.80 (0.66–0.91)	85	0.93 (0.75–1.10)	30	0.036
Global native T1 [950–1050 ms]	1094 (1069–1127)	86	1122 (1076–1160)	30	0.026
Basal septal native T1 [950–1050 ms]	1089 (1054–1119)	86	1114 (1084–1150)	30	0.040
Global native T2 [50–56 ms]	57 (55–60)	82	60 (57–64)	28	0.001
Basal septal native T2 [50–56 ms]	54 (51–58)	82	57(53–61)	28	0.009
Global ECV [25–31%]	56 (49–61)	83	51 (43–60)	25	0.05
Global ECV index	48 (36–59)	83	43 (27–51)	25	0.041
Basal septal ECV [25–31%]	62 (52–68)	83	51 (43–62)	25	0.002
Basal septal ECV index	54 (40–66)	83	42 (28–48)	25	0.001

All data are given as median (interquartile range), if not mentioned otherwise. Units are mentioned in small brackets (). Normal range of values are mentioned in large brackets []. BMI—body mass index; eGFR (CKD-EPI)—estimated glomerular filtration rate according—chronic kidney disease epidemiology collaboration; NYHA—New York Heart Association; NT-proBNP—N-terminal pro brain natriuretic peptides; CMR—cardiovascular magnetic resonance; LV—left ventricle; RV—right ventricle; EF—ejection fraction; EDVi—enddiastolic volume index; ECV—extracellular volume fraction, p < 0.05 is considered as significant; *n* = respective number of patients with available parameters.

**Table 2:** Closer consideration of total intracellular and extracellular myocardial mass and their respective myocardial distribution ratio.

Parameter	ATTRwt	n	AL	n	*p* value
**(A) Total ATTRwt and AL group including both genders**					
Extracellular volume (ECV)	0.56 (0.49–0.61)	83	0.51 (0.43–0.60)	25	0.10
Relative intracellular volume (ICV)	0.44 (0.39–0.51)	83	0.49 (0.43–0.60)	25	0.10
Total intracellular mass (TICM) in g	77.2 (64.9–93.7)	83	71.2 (52.4–90.8)	25	0.21
Total extracellular mass (TECM) in g	127.7 (105.0–159.0)	83	102.5 (72.3–123.4)	25	**0.002**
Ratio TICM / TECM	0.60 (0.49–0.80)	83	0.73 (0.58–1.01)	25	0.10
**(B) Selective consideration of “male” patients**					
Parameter	ATTRwt	n	AL	n	*p* value
Extracellular volume (ECV)	0.57 (0.49–0.61)	73	0.48 (0.40–0.56)	15	**0.007**
Relative intracellular volume (ICV)	0.43 (0.39–0.51)	73	0.52 (0.45–0.60)	15	**0.007**
Total intracellular mass (TICM) in g	81.4 (65.5–96.8)	73	77.1 (70.8–97.7)	15	0.47
Total extracellular mass (TECM) in g	131.9 (113.6–161.4)	73	111.1 (77.0–131.7)	15	**0.02**
Ratio TICM / TECM	0.58 (0.49–0.80)	73	0.83 (0.61–1.16)	15	**0.007**

Significant values are in bold.

## Results

Details of clinical parameters as well as multi-parametric CMR findings are shown in Table 1. As ATTRwt amyloidosis is more common in older male patients, there was a significant difference in age (*p* < 0.001) and gender distribution (*p* < 0.001) between the two groups. Body mass index (BMI) showed a marginal difference (*p* = 0.044) whereas other parameters like eGFR (*p* = 0.59) and clinical assessment of NYHA classification (*p* = 0.34) showed no significant differences between the two groups. Similarly, NT-proBNP serum values were elevated in both groups showing no substantial difference (*p* = 0.33).

### Assessment CMR-based volumetric parameters

**Figure 1:**
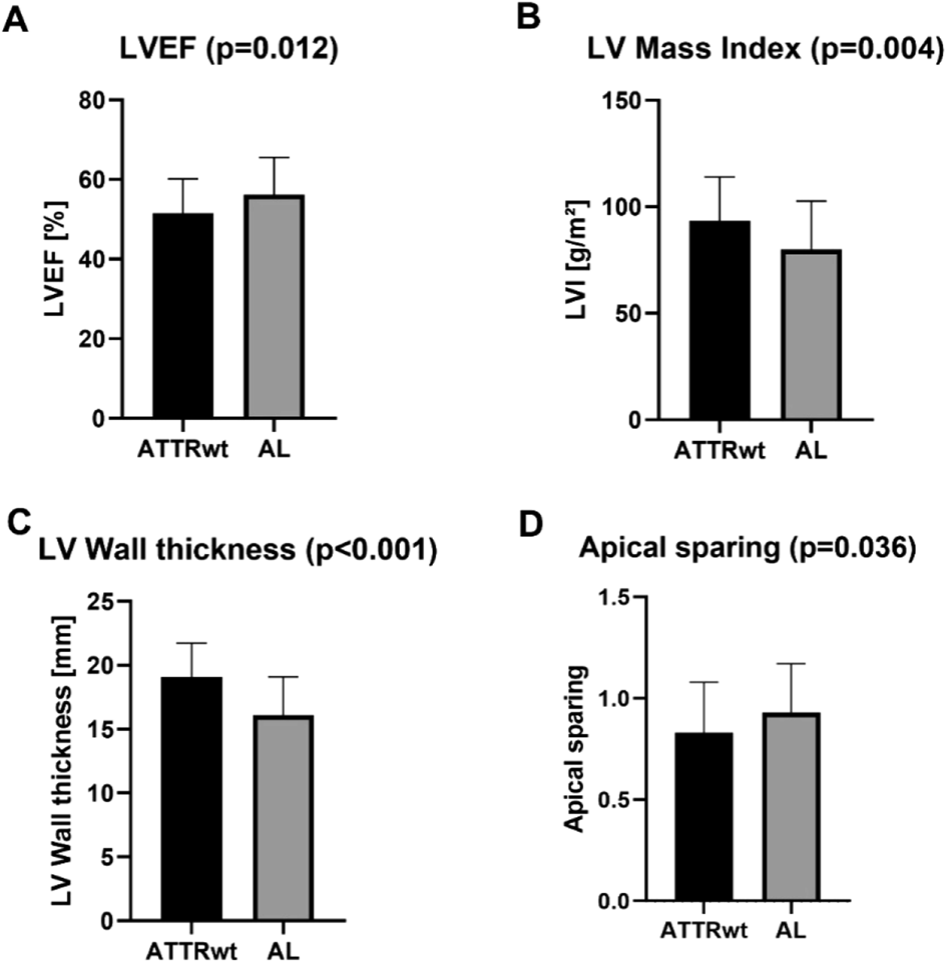
Graph illustrating the differences between ATTRwt and AL amyloidosis group regarding (**A**) left ventricular ejection fraction (LV-EF), (**B**) left ventricular mass index (LVMi), (**C**) left ventricular wall thickness and **(D**) apical sparing; *p*-value < 0.05 is considered as significant.

An overall better cardiac function was observed in the AL group regarding LVEF [52% vs. 58%, *p* = 0.012; Fig. 1A], LV-EDVi [86 ml/m^2^ vs. 75 ml/m^2^, *p* = 0.003] and LV-ESVi [39 ml/m^2^ vs. 32 ml/m^2^, *p* = 0.010]. Similarly, left ventricular mass (LVMi) [91 g/m^2^ vs. 81 g/m^2^, *p* = 0.004; Fig. 1B] and left ventricular wall thickness (LVWT) [19 mm vs. 16 mm, *p* < 0.001; Fig. 1C] were higher in ATTRwt compared to AL. All other CMR volumetric parameters—except from right ventricular ejection fraction (RVEF)—showed similar results like left ventricular parameters as illustrated in Table 1.

### Assessment of CMR-based strain parameters

3D-LV-GLS was reduced in both groups [− 8.0% in ATTRwt vs. − 7.2% in AL] without a statistically relevant difference (*p* = 0.68). The “apical-to-(basal + midventricular)”-ratio of left ventricular regional longitudinal strain (LV-RLS) (reflecting the degree of apical sparing) showed a minor, however, significant difference between both groups [0.80 vs. 0.93, *p* = 0.036] as illustrated in Fig. 1D. The most pronounced impairment of LV-RLS was measured in the basal LV segments in both groups [− 6.6% in ATTRwt vs. − 5.9% in AL; *p* = 0.64].

### Assessment of CMR-based T1-mapping and ECV parameters

**Figure 2:**
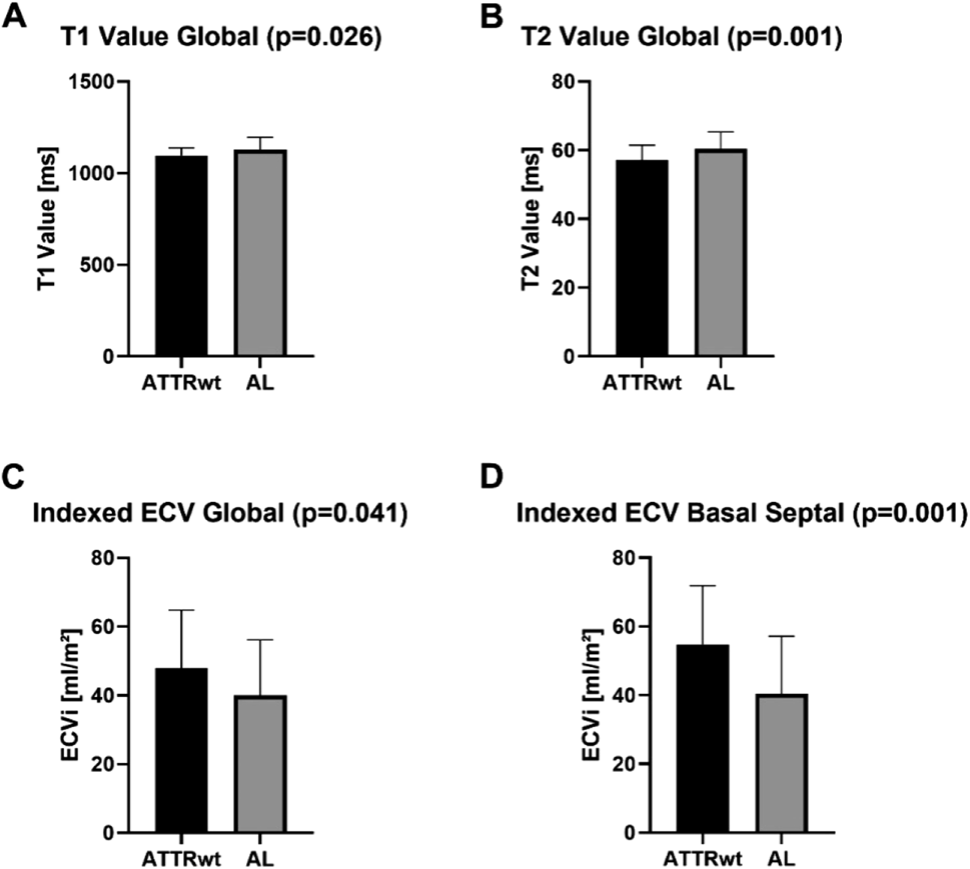
Graph illustrating the differences between ATTRwt and AL amyloidosis group regarding (**A**) global T1-mapping values, (**B**) global T2-mapping values, (**C**) global indexed extracellular volume and (**D**) indexed extracellular volume basal septal; *p*-value < 0.05 is considered as significant.

As illustrated in Table 1, values of native myocardial T1 and T2 were increased in both groups. A comparison of both groups showed significantly higher native T1- and T2-values in AL patients compared to ATTRwt ones: T1 global: 1,094 ms in ATTRwt vs. 1,122 ms in AL (*p* = 0.026; Fig. 2A); T2 global: [57 ms in ATTRwt vs. 60 ms in AL (*p* = 0.001; Fig. 2B).

Accordingly, global myocardial ECV was elevated in both groups [56% in ATTRwt vs. 51% in AL; normal range: 25–31%], with borderline significance (*p* = 0.053). However, when LV mass was taken into consideration, indexed ECV values turned out to be substantially higher in ATTRwt compared to AL [48% vs. 43%, *p* = 0.041, Fig. 2C,D].

### Assessment of total cell volume and relationship between intracellular and extracellular myocardial mass

**Figure 3:**
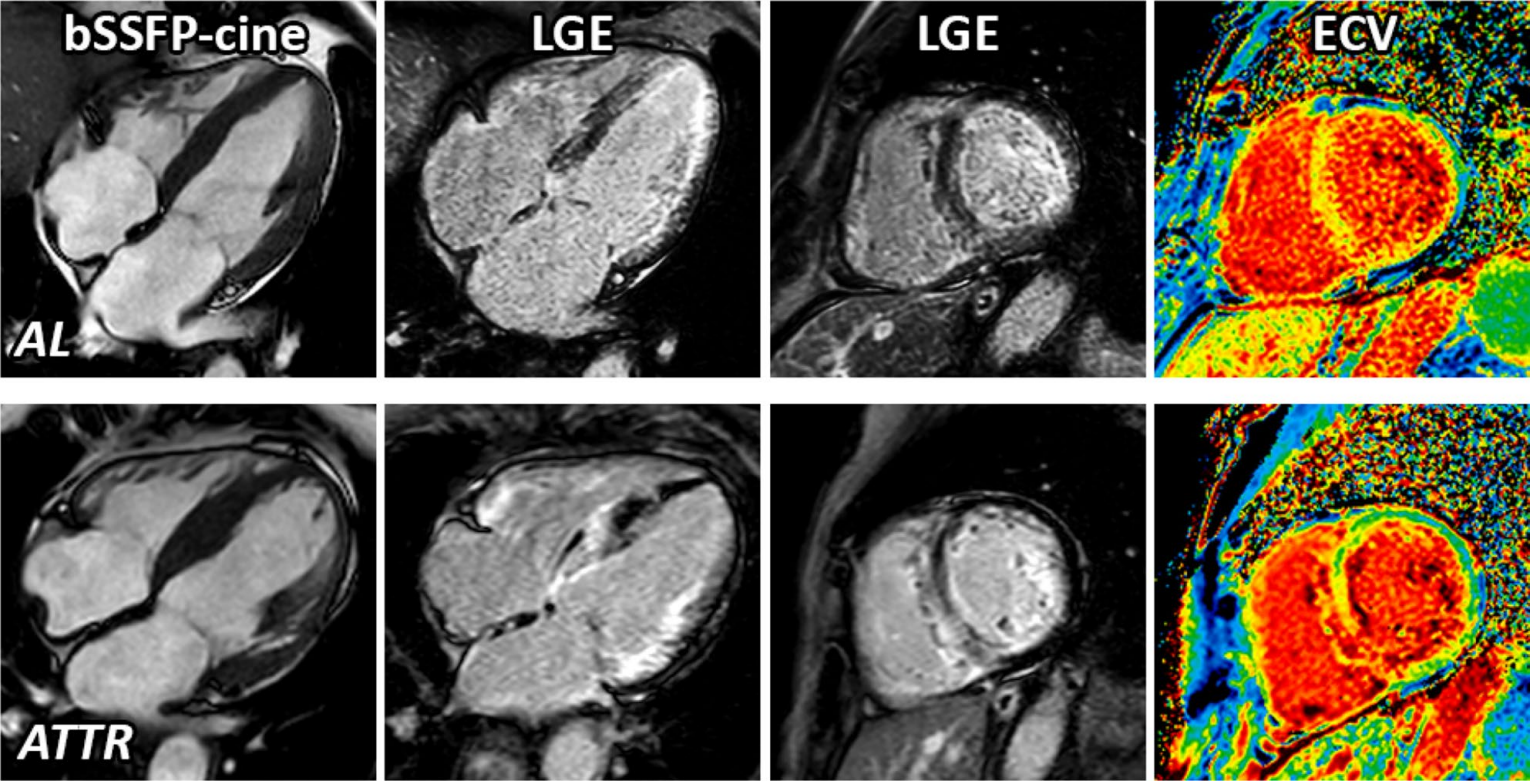
Example of cardiovascular magnetic resonance (CMR) images of AL amyloidosis (upper panel) and ATTRwt (bottom panel) showing cine-images in 4-chamber view in diastole, LGE in 4-chamber view, LGE in short axis as well as corresponding extracellular volume fraction (ECV) maps in a basal short-axis view respectively.

Using the aforementioned global ECV value, we subsequently calculated the relative ICV value as well as absolute TICM and TECM values as illustrated in our methods section. Importantly, when selectively looking at male patients, total myocardial volume (reflected by the aforementioned LV myocardial mass) as well as TECM were substantially higher in ATTRwt compared to AL (131.9 g vs. 111.1 g; *p* = 0.02; Fig. 3), whereas TICM was not significantly different between ATTRwt and AL (81.4 g vs. 77.1 g; *p* = 0.47). Consequently, the ratio [TICM/TECM] was significantly lower in case of ATTRwt when compared to AL (0.58 vs. 0.83; *p* = 0.007; Table 2).

## Discussion

**Figure 4:**
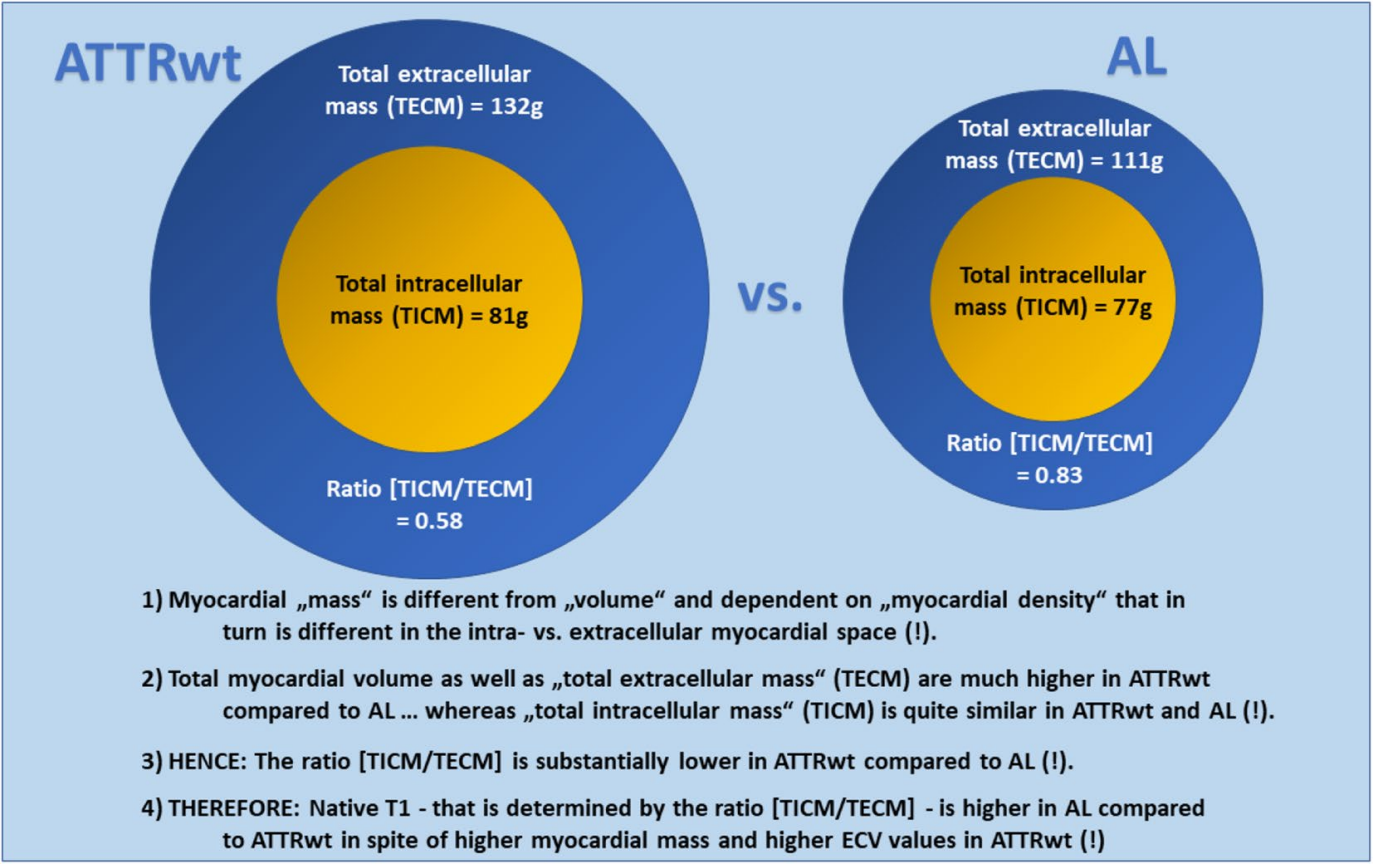
Central illustration of the major study results.

The major results of this study do not only confirm previous data regarding structural differences in cardiac ATTR vs. AL, but also expand our understanding of those essential differences that can be non-invasively assessed by multi-parametric CMR: (1) The present data confirm the presence of the “native T1 vs. ECV paradox” in cardiac ATTR compared to AL, since a lower myocardial native T1-value was observed in ATTRwt patients—in spite of higher myocardial mass and higher global ECV-values—compared to AL patients. (2) A more detailed analysis of “intracellular” and “extracellular” myocardial mass revealed that total myocardial mass as well as “total extracellular mass” (TECM) were substantially higher in ATTRwt compared to AL, whereas “total intracellular mass” (TICM) was not significantly different between ATTRwt and AL. Consequently, the ratio [TICM/TECM]—which decisively determines the extent in change of native T1—was significantly lower in case of ATTRwt when compared to AL (Fig. 4).

### The "native T1 vs. ECV paradox" in cardiac ATTR vs. AL amyloidosis

As discussed in detail previously^17^, the London group published several studies addressing the use of T1-mapping and ECV measurement (in order to depict structural changes) in patients with cardiac amyloidosis in the last years^7,9,10,12,13^. Based on this groups data regarding native T1 and LV mass in ATTR vs. AL, a "native T1 vs. ECV paradox" emerged in CA patients. Noteworthy, our present data are in line with the aforementioned CMR data from the London group and confirm the presence of the “native T1 vs. ECV paradox” in cardiac ATTR compared to AL: lower myocardial native T1 values were observed in ATTRwt—in spite of higher myocardial mass and higher global ECV values—compared to AL.

In order to address this important question, we need to recapitulate some methodological aspects of CMR-based volume/mass quantification. “Volumetric” analyses in CMR are usually performed based on cine-CMR images by contouring of endocardial as well as epicardial LV myocardial borders. After receiving a 3D myocardial “volume” of the LV, the respective software uses a pre-specified “myocardial density” factor (usually 1.055 g/ml) in order to calculate LV “mass” based on the aforementioned 3D volume. Importantly, this approach does not consider any potential differences in “myocardial density”, caused by e.g. the presence of specific cardiomyopathies (such as cardiac amyloidosis) … nor does it account for any differences in myocardial density between the intracellular vs. extracellular space of the myocardium. Since amyloid fibrils accumulate in the “extracellular” space of the myocardium only, the undifferentiated use of the aforementioned myocardial density factor of “1.055 g/ml” for volume-based calculation of myocardial mass is not appropriate. Therefore, we tried to address this issue by a different approach …

### Compartment-specific myocardial density—an essential, however, overlooked issue

First, we need to address the important issue of myocardial structure and the relationship between “composition” and “density”. Unfortunately, detailed data regarding the relationship between myocardial composition—with a specific focus on extracellular vs. intracellular compartments—and compartment-specific myocardial density are still missing (not only in case of cardiac amyloidosis)^26^. However, a detailed and convincing estimate of myocardial tissue composition based on pre-established laws for density, volume and compartment composition does already exist^23^. According to this model, the major three constituents that affect individual compartment/tissue density are a) proteins (+ solutes), b) water and c) fat—and of course their distribution and relationship. In case of a “normal” myocardial composition, the respective tissue density is around 1.053 g/ml according to this model—which is convincingly close to the aforementioned value of 1.055 g/ml that is known for human myocardium. However, if the percentage (concentration) of proteins (+ solutes) increases in this model (due to e.g. dehydration or due to e.g. amyloid deposition), then the respective compartment density also increases and approaches values of up to 1.40 g/ml. This substantial change in tissue density needs to be considered when we are approaching an “infiltrative” cardiac disease such as cardiac amyloidosis.

### Our novel approach in solving the "native T1 vs. ECV paradox"

T1-mapping based measurement of global myocardial ECV allows to calculate the extent of myocardial ICV by the simple formula ICV = [1–ECV]. Importantly, the aforementioned calculation of the myocardial ECV proportion (and subsequent ICV proportion) is based on pre- and post-contrast T1-maps and therefore not dependent on volume- or mass-based analyses. Thereafter, we defined “total intracellular mass” (TICM) of the myocardium by the formula TICM = [ICV × total LV myocardial volume × myocardial density]. In this context, the parameter “total LV myocardial volume” was correctly derived from our volumetric short-axis cine-stack analysis. Similarly, “total extracellular mass” (TECM) was defined by the formula TECM = [ECV × total LV myocardial volume × myocardial density]. Such a detailed approach for analyzing TICM and TECM allowed us to further consider structural differences between the intra- vs. extracellular space based on the individual “myocardial density factor”. Taking into account some minor hypothetical inaccuracy, we supposed that structural changes in case of cardiac amyloidosis primarily take place in the extracellular space—but not in the intracellular space—since amyloid fibrils only accumulate in the extracellular compartment. Therefore, we used the well-known myocardial density value for humans of 1.055 g/ml when calculating TICM. In contrast, when calculating TECM, we used a different myocardial density value of 1.38 g/ml as a best estimate, since this value is expected to be more appropriate in case of extracellular “protein” accumulation^23^.

Using such a detached approach for quantification of TICM and TECM allowed us to subsequently calculate the ratio between TICM and TECM for ATTRwt and AL that turned out to be “lower” in case of ATTRwt compared to AL in spite of higher myocardial mass and higher global ECV values in ATTRwt. And since this ratio [TICM/TECM] is expected to decisively determine the extent in change of native T1, we may explain the “native T1 vs. ECV paradox” in cardiac amyloidosis (relatively lower native T1 values in case of ATTRwt) by the aforementioned lower ratio [TICM/TECM] in ATTRwt compared to AL. In other words, since amyloid deposits are particularly dense (and presumably denser than collagen-based fibrosis), a greater deposition in the extracellular compartment (supposed in case of ATTRwt due to higher TECM) is associated with a relatively lower increase in T1, since T1 is driven by the relative extent of TICM in comparison to TECM—if the extracellular free water content is relatively reduced by dense and insoluble amyloid deposits (whereas the intracellular free water content should rather stay untouched).

### And not to forget the T2-mapping results

From a simplified pathophysiological point-of-view, cardiac AL is different from cardiac ATTR and not only characterized by “extracellular” accumulation of (light-chain) amyloid fibrils, but also by toxic effects that are caused by those light-chains, subsequently leading to myocardial inflammation as well as intra- and extracellular edema (including myocyte swelling). Hence, in case of “active” cardiac AL, we expect a predominance of the aforementioned toxic effects in addition to extracellular light-chain accumulation. Consequently, these effects should also result in “increased” native T2 values in case of AL when compared to ATTRwt. Noteworthy, our present findings of increased myocardial T2 values in case of AL (compared to ATTRwt) are in line with previous data from the London group^27^, but contradict recent data from Cuddy et al.^27^. Obviously, more extensive multi-center data are strongly required to better assess this important issue.

### Clinical value and outlook

Today, CMR does not only play an important clinical role for diagnosis of CA, but also for therapy monitoring since specific therapeutic options (being mostly highly expensive) are available today. Obviously, we need to (a) better understand the complex interplay between amyloid deposits in the extracellular myocardial space and subsequent changes in myocardial function … and (b) to better monitor such structural and functional changes—if possible at the cellular level. In this context, our present data illustrate that multi-parametric CMR does not only allow to accurately assess cardiac function in patients with CA, but also allows to accurately assess and monitor structural changes in the extracellular as well as intracellular compartment of these patients by considering disease-specific particularities regarding compartment-based “composition” and “density”. Such detailed information may play a decisive role when assessing the individual cardiac disease course and monitoring therapy response when approaching an “infiltrative” cardiac disease such as CA. Obviously, future studies addressing compartment-specific myocardial tissue densities are strongly required in order to better assess and understand this important issue.

### Study limitations

Obviously, the myocardial density factor of 1.38 g/ml that we used for calculation of TECM was not validated by any histopathological analyses within this study, since this was—unfortunately—technically not possible. This definitely needs to be done—if possible—within the scope of future studies. We discussed this issue in detail with our collaborating pathologists; unfortunately, an easy approach to determine extracellular vs. intracellular myocardial density based on biopsy samples does not exist so far. However, even if the respective myocardial density factor for the extracellular space in case of CA will be somewhat lower than 1.38 g/ml, we are convinced that the respective value (in advanced CA) will be much higher than 1.055 g/ml which is the well-known and mostly used myocardial density value for humans. Moreover, our AL group was rather small (N = 30 patients) since we only included those AL patients who were not treated with any disease specific therapy prior to our CMR study. Finally, future studies need to carefully assess possible gender differences regarding the findings of the present study.

## Conclusions

Our data confirm the presence of a “native T1 vs. ECV paradox” with lower native T1 values in spite of higher myocardial mass and ECV values in ATTRwt compared to AL. Importantly, this observation may be explained by a lower TICM/TECM “ratio” in case of ATTRwt compared to AL—since native T1 is determined by this ratio. Since non-invasive imaging methods such as CMR-based mapping are not only used for the diagnosis of cardiac amyloidosis, but also enable non-invasive monitoring of cardiac amyloidosis progression (or regression), a better understanding of mapping-based CMR results and of myocardial structure (with a particular focus on compartment-based “composition” and “density”) is crucial.

## Data Availability

The datasets used and/or analysed during the current study are available from the corresponding author on reasonable request.

## Abbreviations

ATTRwt: Wild-type transthyretin amyloidosis
CM: Cardiomyopathy
CMR: Cardiovascular magnetic resonance
LV-EF: Left ventricular ejection fraction
ECV: Extracellular volume fraction
LVMi: Left ventricular mass index
LVWT: Left ventricular wall thickness
LGE: Late-gadolinium-enhancement
NT-proBNP: N-terminal pro brain natriuretic peptides
eGFR: Estimated glomerular filtration rate
NYHA: New York Heart Association
LV-GLS: Left ventricular global longitudinal strain
RVEF: Right ventricular ejection fraction
RVEDVi: Right ventricular enddiastolic volume index
